# The role of circRNAs and miRNAs in drug resistance and targeted therapy responses in breast cancer

**DOI:** 10.20517/cdr.2024.62

**Published:** 2024-08-20

**Authors:** Meilan Zhang, Zhaokuan Zheng, Shouliang Wang, Ruihan Liu, Mengli Zhang, Zhiyun Guo, Hao Wang, Weige Tan

**Affiliations:** ^1^The Affiliated Panyu Central Hospital of Guangzhou Medical University, Guangzhou 511400, Guangdong, China.; ^2^Department of Orthopedics, Affiliated Huadu Hospital, Southern Medical University (People’s Hospital of HuaduDistrict), Guangzhou 510810, Guangdong, China.; ^3^Department of Breast Surgery, the First Affiliated Hospital, Guangzhou Medical University, Guangzhou 510120, Guangdong, China.

**Keywords:** miRNAs, circRNAs, breast cancer, clinical values

## Abstract

MicroRNAs (miRNAs) are small non-coding RNAs comprising 19-24 nucleotides that indirectly control gene expression. In contrast to other non-coding RNAs (ncRNAs), circular RNAs (circRNAs) are defined by their covalently closed loops, forming covalent bonds between the 3’ and 5’ ends. circRNAs regulate gene expression by interacting with miRNAs at transcriptional or post-transcriptional levels. Accordingly, circRNAs and miRNAs control many biological events related to cancer, including cell proliferation, metabolism, cell cycle, and apoptosis. Both circRNAs and miRNAs are involved in the pathogenesis of diseases, such as breast cancer. This review focuses on the latest discoveries on dysregulated circRNAs and miRNAs related to breast cancer, highlighting their potential as biomarkers for clinical diagnosis, prognosis, and chemotherapy response.

## INTRODUCTION

Breast cancer (BC) is the most common cancer in women worldwide and the leading cause of cancer-related death. In 2022, nearly 2.3 million new cases of BC were diagnosed globally^[1]^. There are many factors that affect the incidence of BC, including age, early menarche, late menopause, hormone-replacement therapy, lifestyle factors (e.g., alcohol intake and physical inactivity), BRCA1 and BRCA2 mutations, and so on. The signaling pathways recognized to be involved in BC are steroid hormone signaling, human epidermal growth factor receptor 2 (HER2/ERBB2) signaling pathway, cyclin D1/cyclin-dependent kinase 4/6/retinoblastoma protein (cyclin D1/CDK4/6/RB1), phosphatidylinositol 3-kinase/protein kinase B (PI3K-AKT) pathway, mitogen-activated protein kinase (MAPK) signaling pathway, and progranulin (PGRN) signaling pathway^[2-4]^. Based on the expression of estrogen receptor (ER), progesterone receptor (PR), and HER2, BC is categorized into four molecular subtypes: Luminal A, Luminal B, HER2-positive, and triple-negative^[2]^. With the wide availability of mammography screening and targeted treatment of different molecular subtypes, the survival rate of BC patients has significantly improved over the past two decades. Unfortunately, the classic parameters currently used to guide treatment decisions remain imperfect, especially in advanced cancers, which eventually develop drug resistance. Therefore, it is urgent to find biomarkers for early detection of BC and prediction of response to treatment, thereby improving the selection of optimized treatment.

miRNAs are 19-24-nucleotide-long non-coding RNAs (ncRNAs) that are processed from hairpin precursors^[5]^. These small non-coding RNAs (snRNAs) are pivotal in regulating gene expression by operating at the post-transcriptional level to impact protein synthesis^[6,7]^. They mediate the silencing process of approximately 30% of coding genes after transcription^[8]^. miRNAs widely participate in regulating important biological processes such as proliferation, apoptosis, differentiation, immune response, and maintenance of cell or tissue specificity^[9,10]^. miRNAs can be used as biomarkers for cancer diagnosis and the prediction of curative effects^[11,12]^.

circRNAs, a subclass of ncRNAs, constitute a covalently closed circular structure in eukaryotes. They are transcribed from protein-coding genes and undergo an unconventional precursor messenger RNA (pre-mRNA) splicing process known as back splicing. This intricate mechanism involves the ligation of the 3’-end of an exon to the 5’-end donor splice site of the same exon or an upstream exon^[13]^. Structurally, circRNAs, originating from diverse biogenesis pathways, can be categorized into circularized intronic, exon-intronic, exonic, and transfer RNA (tRNA) intronic subtypes^[14]^. During circRNA biogenesis, distant donor and acceptor splice sites must be brought close to facilitate ligation, culminating in the formation of a circular structure. This process is orchestrated by various mechanisms^[15]^. Several studies have shown that circRNA affects the progression of BC^[16-18]^.

This review highlights the latest discoveries related to circRNAs and miRNAs associated with BC, emphasizing their potential as biomarkers for clinical diagnosis, prognosis, and response to chemotherapy. By comprehensively exploring the roles of circRNAs and miRNAs in BC, we aim to deepen our understanding of this disease and provide insights for improving diagnostic and therapeutic strategies.

## BIOGENESIS AND FUNCTIONAL MECHANISM OF MIRNAS AND CIRCRNAS

**Figure 1:**
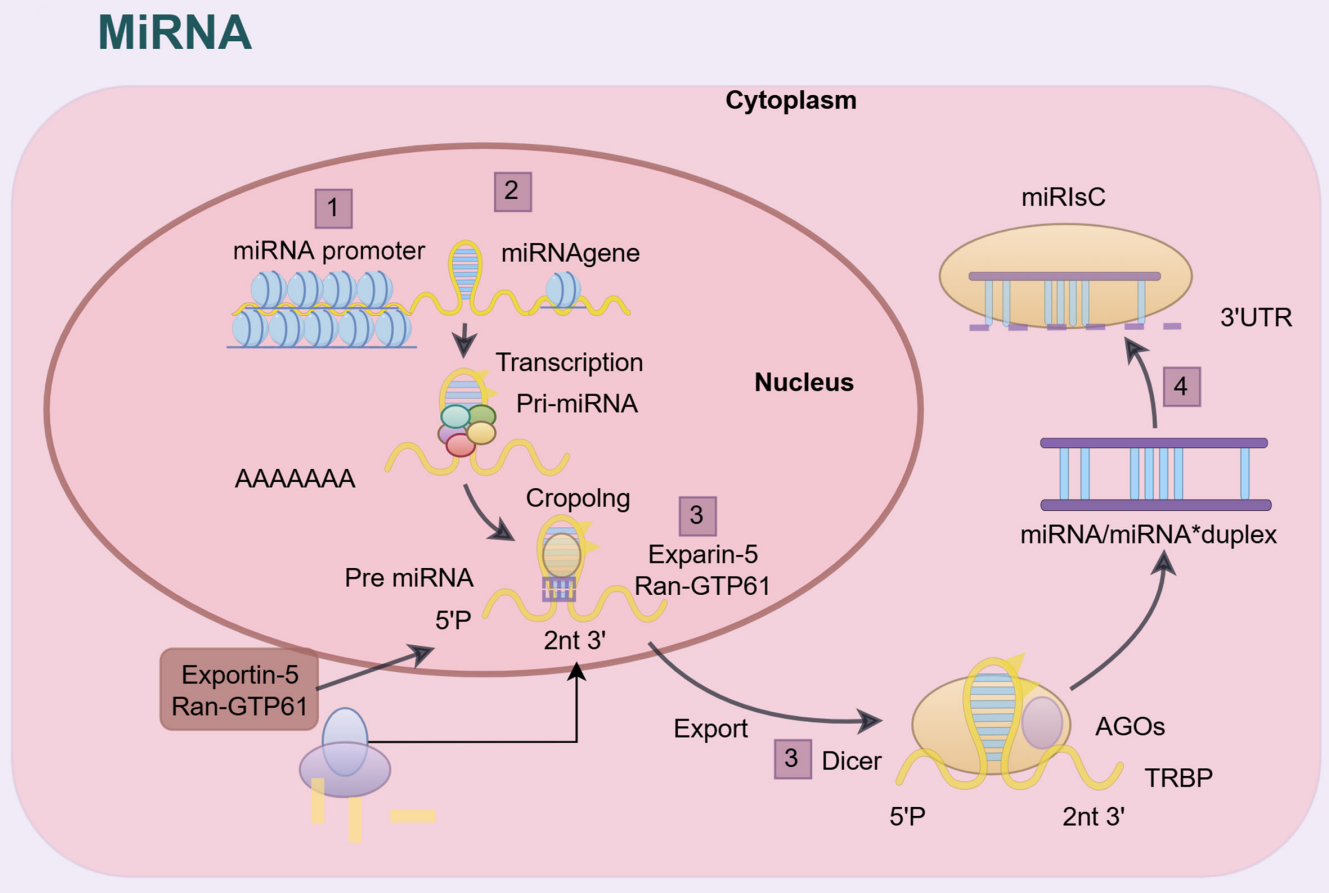
Biogenesis and functional mechanism of miRNA. MiRNA is transcribed from DNA into primary miRNA (pri-miRNA) transcripts by RNA polymerase II or III in the cell nucleus. Subsequently, pri-miRNA is cleaved into precursor miRNA (pre-miRNA). Under the regulation of RanGTP protein, pre-miRNA is transported to the cytoplasm through the shuttle protein exportin 5. After entering the cytoplasm, Dicer RNase catalyzes the cleavage of pre-miRNA into mature double-stranded miRNA. One of the miRNA single strands binds to RISC (also known as miRISC). RISC: RNA-induced silencing complex.

miRNA molecule belongs to a kind of snRNA encoded by eukaryotic genomic DNA, which is evolutionarily highly conserved. The biosynthesis of miRNA is catalyzed by multiple enzymes in different cell compartments, which is a complicated process. Initially, miRNA is transcribed from DNA into primary miRNA (pri-miRNA) transcripts by RNA polymerase II or III in the cell nucleus. Afterwards, pri-miRNA undergoes cleavage by the microprocessor complex, comprising RNAse III Drosha and its cofactor DiGeorge syndrome critical region 8 (DGCR8), resulting in the formation of a precursor miRNA (pre-miRNA) hairpin structure. Under the regulation of the Ras superfamily member RanGTP protein, pre-miRNA is transported to the cytoplasm via the shuttle protein exportin 5^[19]^. Once in the cytoplasm, the ribonuclease Dicer RNase catalyzes the cleavage of pre-miRNA into mature double-stranded miRNA with a length of 21 to 23 nucleotides^[20]^. After being processed by Dicer, the double-stranded miRNA becomes two single strands known as the guide strand or mature miRNA. One of the two strands binds to RNA-induced silencing complex (RISC, also referred to as miRISC), and the other strand is degraded and is known as the star strand miRNA or passenger strand^[21,22]^. The binding mechanism between mature miRNA and RISC to regulate target mRNA contains: (1) mRNA cleavage by miRNA binding site; and (2) translation inhibition. Pairing between miRNA and typically 3’ untranslated region (3’- UTR) of target mRNA is crucial for their interaction in animals^[23]^. Each miRNA may act on multiple mRNAs, and conversely, an mRNA can be regulated by several different miRNAs [Figure 1]^[24,25]^.

**Figure 2:**
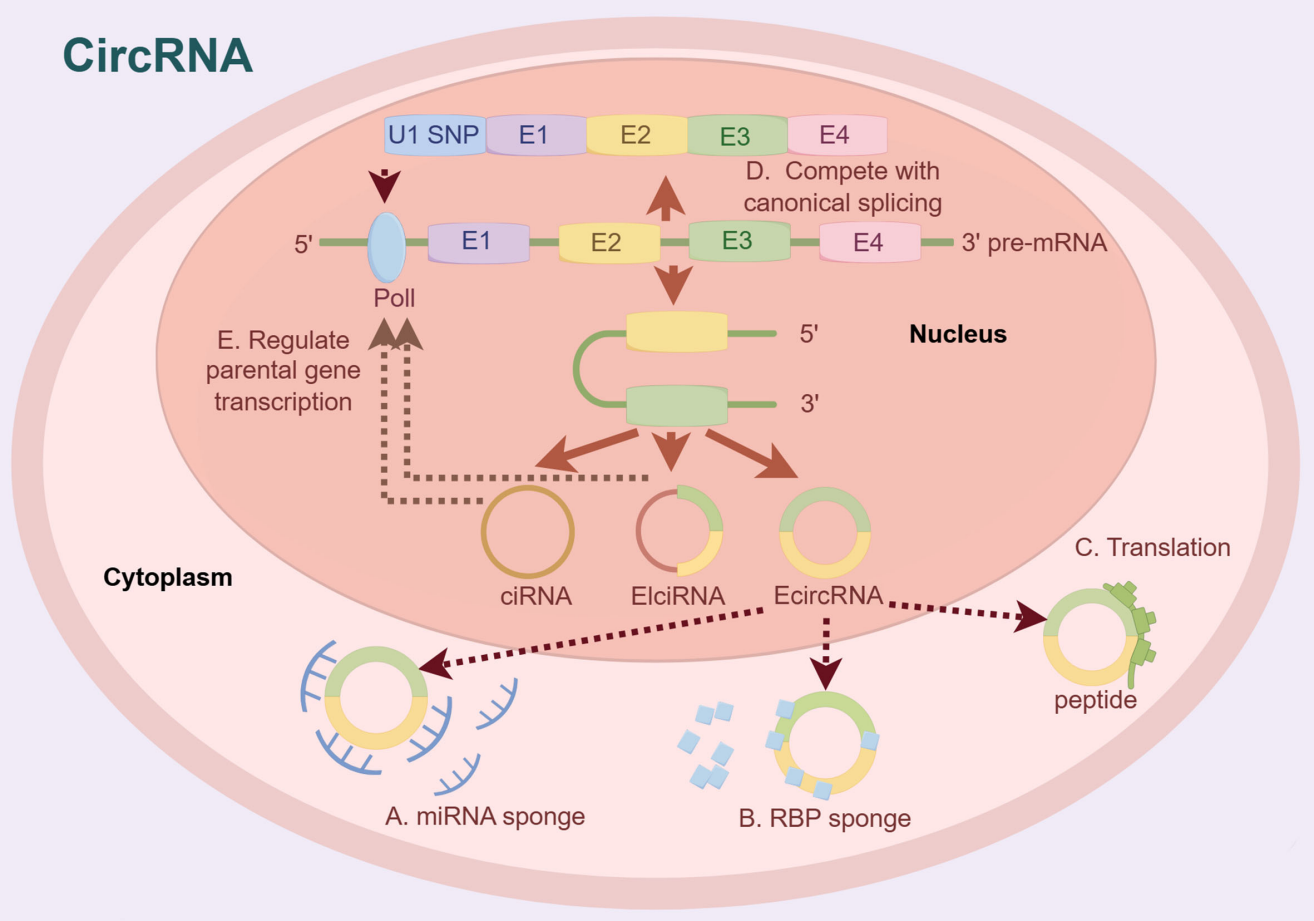
Biogenesis and functional mechanism of circRNA. First, circRNA is transcribed from genomic DNA by RNA polymerase II or III. Subsequently, during the splicing process, the downstream 5’ splice site of the precursor mRNA (pre-mRNA) is back-spliced with the upstream 3’ splice site to form a circular molecule.

Circular RNA (circRNA) is a non-coding RNA characterized by a covalently closed loop structure devoid of a 5’ cap and a 3’ polyadenylated tail. First, circRNA is transcribed from genomic DNA by RNA polymerase II or III, similar to linear mRNA transcripts. Subsequently, during the splicing process, the downstream 5’ splice site of the precursor mRNA (pre-mRNA) is back-spliced with the upstream 3’ splice site to form a circular molecule^[26-28]^. CircRNAs can significantly contribute to the progression of diverse diseases by exerting various biological effects^[29-31]^. In the context of known tumor development, circRNA exhibits multiple functions, including: (A) acting as a sponge for competing endogenous RNA (ceRNA) or miRNA; (B) acting as sponges for RNA-binding proteins (RBPs); (C) participating in protein translation; (D) regulating transcription and splicing; and (E) interacting with RNA-binding proteins [Figure 2]^[32-36]^.

## MIRNAS AND CIRCRNAS AS DIAGNOSTIC MARKERS IN BC

BC is an epithelial malignancy arising from the terminal ductal lobular unit of the breast, which is generally categorized into non-invasive or invasive carcinoma based on histology. Non-invasive carcinoma is further divided into ductal and lobular carcinoma *in situ*. Invasive BC penetrates surrounding breast tissue and primarily includes invasive lobular carcinoma (ILC) and invasive ductal carcinoma (IDC)^[2]^. BCs in different tissue types and molecular subtypes differ in pathology, genomic profiles, metastatic organ chemotaxis, and responses to treatment. But in any case, early diagnosis of BC is the most effective way to reduce mortality. Identifying biomarkers for the early detection of BC is crucial.

**Figure 3:**
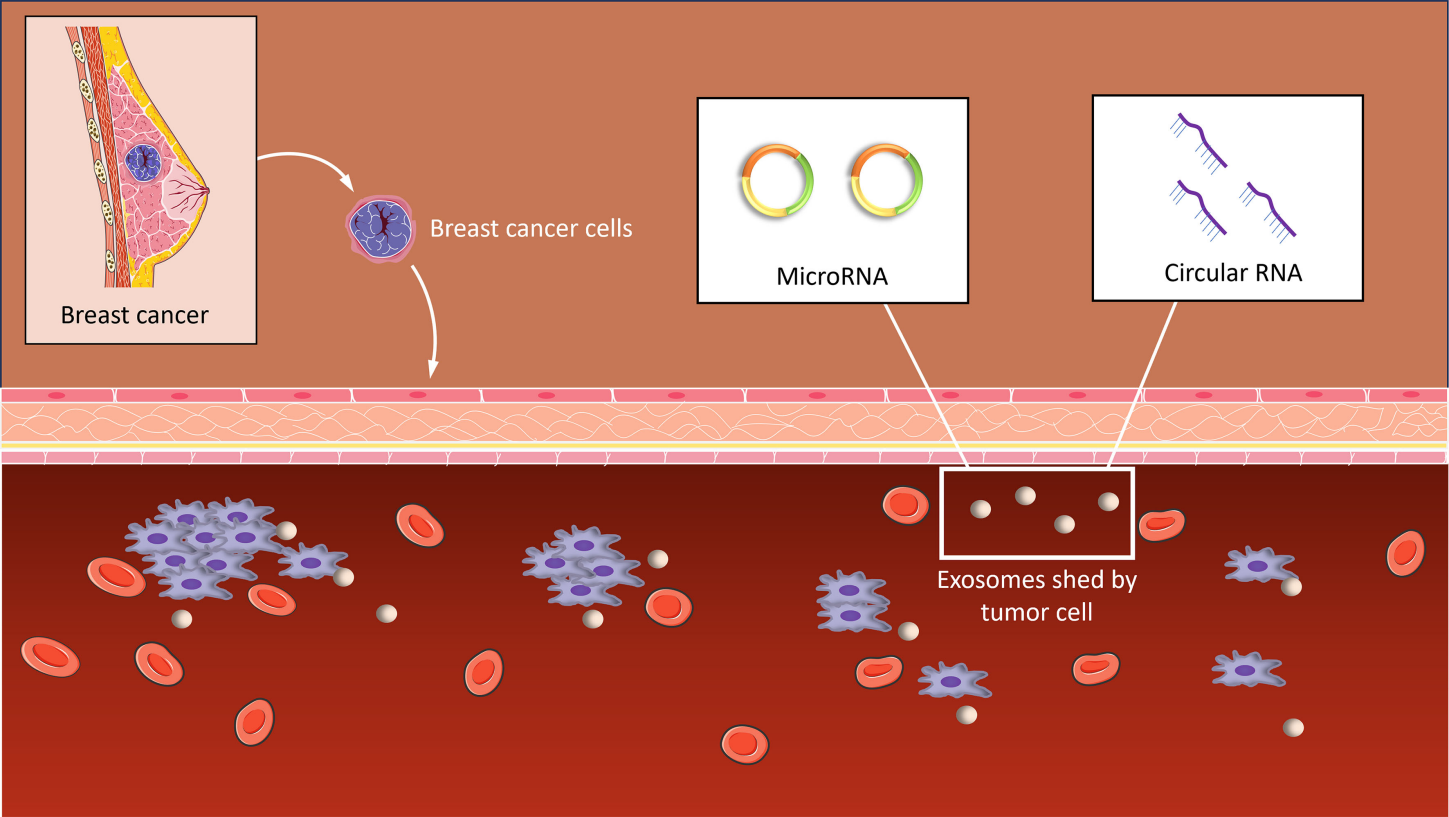
CircRNAs and miRNAs in the exosomes of breast cancer patients. Some circRNAs and miRNAs can be detected in the serum exosomes of breast cancer patients, and analysis of exosomes in serum can provide information about the disease status.

Since miRNAs and circRNAs control many biological events related to cancer, they are often associated with the early diagnosis of the disease. Studies have shown that some circRNAs and miRNAs exhibit differential expression patterns in BC compared to normal tissues, and these circRNAs and miRNAs may be involved in the occurrence and development of BC by regulating cancer-related genes. Similarly, certain circRNAs and miRNAs can also be detected in serum exosomes, and their expression levels may be associated with clinical features of BC [Figure 3]. Recent research has found that five plasma miRNAs (miR-122-5p, miR-210-3p, miR-146b-5p, let-7b-5p, and miR-215-5p) exhibited significantly different expression levels between BC patients and normal controls^[37]^. A signature comprising the five miRNAs showed excellent discriminatory accuracy with the area under curve (AUC) values 0.683, 0.966, and 0.978 for the training, testing, and external validation sets, respectively. When combined, twelve cell-free miRNAs showed outstanding discriminatory accuracy, achieving an AUC value of 0.95. Furthermore, the expression of circ_0001640, circ_0001531, and circ_0000745 were upregulated in BC patients compared with benign tumors and healthy controls. The AUC of the panel was 0.9130^[38]^. These results indicate that miRNA and circRNA can be used as markers for the diagnosis of BC. Further studies have found that miRNAs exhibit different expression levels in BCs of various tissue types. A comparison of miRNAs between low-grade and high-grade BCs revealed that 25 miRNAs were significantly upregulated while 18 miRNAs were significantly downregulated in high-grade cases^[39]^. Additionally, 107 miRNAs were identified as potential biomarkers for stratifying different types, grades, and stages of IDC^[40]^.

The continuous discovery in research also indicates that miRNA and circRNA expression influence proliferation, invasion, metastasis, and epithelial-mesenchymal transition (EMT) in BC. Compared to healthy controls, the expression levels of miR-144-3p, miR-23a-3p, miR-148a-3p, miR-130a-5p, and miR-152-3p were lower in BC patients, which was associated with clinical stage and lymph node metastasis^[41]^. Overexpression of circRHOT1 and circ_0076611 enhanced the proliferative ability of triple-negative breast cancer (TNBC) cells and promoted the proliferation, invasion, metastasis, and EMT of BC^[42-44]^. In addition, miRNA and circRNA combined with other diagnostic markers can improve the sensitivity of BC diagnosis. MiR-1910-3p in serum exosomes may be used as an effective diagnostic marker for BC diagnosis, and when combined with CA153, it can improve the sensitivity of BC diagnosis^[45]^. A combination of two miRNAs, miR-4710 and miR-629-3p, along with three clinicopathologic factors, lymphovascular invasion, ultrasound findings, and T stage, showed an AUC of 0.86, which may help diagnose ALN metastasis prior to surgery in a less-invasive manner than SLNB^[46]^.

However, there is still controversy about the application of miRNA in diagnosis. Several studies have found that serum miR-21 expression is abnormally elevated in BC. By comparing miR-21 expression levels in the plasma of 252 BC patients, 82 benign breast tumors, and 127 healthy controls, plasma miR-21 levels were found elevated in BC patients, which were significantly decreased after surgery compared with pre-operation. The results indicate that plasma miR-21 level is a crucial biomarker for BC diagnosis^[47]^. Additionally, the level of miR-21 in serum exosomes of BC patients with bone metastasis is significantly elevated compared to other subgroups, reflecting the significance of miR-21 as a potential target for clinical diagnosis of BC bone metastasis^[48]^. In contrast, a publication reported that whole blood miR-21 concentration did not differ among pre-adjuvant therapy, post-adjuvant therapy patients, and healthy individuals^[49]^. In addition, plasma miR-21 concentration did not directly reflect tumor expression^[49]^. These findings reveal the lack of consistency and reproducibility in current studies of miRNA as biomarkers. The reasons for these differences may be related to the differences in miRNA expression in different sample types, the use of different sample collection and processing schemes, and study designs. Research on circRNAs also faces the same issues. In summary, research on miRNA and circRNA as BC biomarkers still faces the obstacles of inconsistency and irreproducibility. To apply miRNA and circRNA as practical biomarkers in clinical practice, a standardized set of scientific detection methods needs to be established. The integration of classical detection methods for multiple miRNAs and circRNAs may help improve diagnostic capabilities.

## MIRNAS AND CIRCRNAS AS PROGNOSTIC AND PREDICTIVE BIOMARKERS IN BC

Studies on circRNA and miRNA in BC tissue and serum samples have shown that the expression patterns of these molecules are associated with tumor malignancy and poor prognosis. Analysis of circRNA and miRNA in serum samples can provide a non-invasive method for the prognostic assessment of BC patients.

Several studies have evaluated the relationship between miRNA expression and BC survival or prognostic prediction. High levels of miR-421, miR-128-1, and miR-128-2 were significantly associated with adverse clinical features and cancer recurrence, and can serve as potential prognostic markers for BC^[50]^. High expression of miR-205, miR-133a, miR-21, miR-155, and miR-92b-3p were correlated with poor overall survival in BC patients^[51]^. Conversely, high expression of some miRNAs can improve the overall survival of BC patients. For example, high expression of miR-451a and miR-367 can improve progression-free survival and overall survival in BC patients^[52,53]^. In addition, miRNA expression may also predict the effects of certain chemotherapeutic drugs and antitumor immune responses. A combination of eight serum miRNAs, (miR-3160-5p, miR-5698, miR-4710, miR-4483, miR-575, miR-8089, miR-296-3p, and miR-4755-3p) was established as biomarkers to predict the responsiveness to eribulin and the emergence of new distant metastases in metastatic BC patients^[54]^. Bioinformatic analysis of human BC databases revealed that high serum and tumor miR-155 levels were correlated with favorable antitumor immune profiles and better patient outcomes, which may be a favorable prognostic marker for BC patients^[55]^. Although the above-mentioned miRNAs as predictive markers have not yet been used in clinical applications, they provide sufficient evidence for the use of miRNAs as prognostic markers for BC.

Similar to miRNA, there is also a relationship between circRNA expression and BC survival or prognosis. Zhong *et al*. collected serum samples from 45 BC patients and 45 normal individuals for qRT-PCR analysis and found that the expression of circRASSF2 was significantly increased in the BC group^[56]^. CircRASSF2 acts as a sponge for miR-1205 and regulates the expression of HOXA1, thereby promoting BC proliferation, migration, and invasion. BC patients with high circRASSF2 expression have lower overall survival and progression-free survival compared to those with low circRASSF2 expression. The expression of circCDYL is upregulated in tumor tissue and serum of BC patients, and its upregulation is associated with a higher tumor burden, shorter survival, and poor response to clinical treatment^[57]^.

## MIRNAS, CIRCRNAS, AND DRUG RESISTANCE IN BC

In the treatment of malignant tumors, chemotherapy is one of the commonly used methods and has a broad prospect for future development. However, due to the emergence of drug resistance, the therapeutic effect of chemotherapy often fails to reach expectations. The mechanisms leading to drug resistance include changes in the cell cycle, enhanced DNA repair, functional changes in the cell death mechanism, increased drug efflux, and EMT. With the advancement of DNA and RNA microarray and sequencing technologies, extensive studies have demonstrated that miRNA and circRNA play a vital role in drug resistance. A comprehensive understanding of the role of miRNA and circRNA in the molecular mechanisms leading to drug resistance will help to develop better strategies for cancer treatment.

The cell cycle encompasses the entirety of processes that a cell undergoes from the conclusion of one division to the conclusion of the subsequent division, and consists of four consecutive phases: G0/G1 (Gap 0/1), S (synthesis), G2 (Gap 2), and M (mitosis)^[58]^. Disruption of the cell cycle is a well-known hallmark of cancer, and its abnormal activation is associated with drug resistance. Quite a few miRNAs have been found to regulate genes involved in the cell cycle, leading to drug resistance. Bao *et al*. found low expression of miR-93 in paclitaxel (PTX)-resistant BC samples compared to PTX-responsive patients^[59]^. This is because miR-93 inhibits the pRB/E2F1 pathway and AKT phosphorylation by directly targeting E2F1 and CCND1, which inhibits cell proliferation and cell cycle progression to enhance the therapeutic effect of PTX *in vivo*. In addition, E2F1 has been identified as a direct target of miR-302b, which enhances sensitivity to cisplatin by regulating the E2F1/ATM axis^[60]^.

At present, most chemotherapeutic agents induce direct or indirect DNA damage via double-strand breaks (DSB). DNA damage can be repaired through homologous recombination (HR) or non-homologous end-joining (NHEJ)^[61]^. Enhanced DNA damage repair response (DDRR) will lead to the development of drug resistance. Therefore, the regulation of DDRR-related genes by miRNA can affect drug sensitivity. Flap endonuclease 1 (FEN1) participates in various DNA repair pathways. MiR-140 suppresses FEN1 expression by directly binding to its 3’ untranslated region, which results in impaired DNA repair^[62]^. The low expression of MiR-140 will lead to the resistance of doxorubicin. In addition, miR-30c has been implicated in adriamycin (ADM) response. MiR-30c targets the DNA repair proteins Fanconi anemia complementation group F protein (FANCF) and DNA polymerase REV1 (REV1) protein, thereby sensitizing the cells to ADM^[63]^. In p53-mutant BC, mutations in p53 result in decreased miR-30c expression, leading to ADM resistance.

Cell death is crucial in multiple physiological functions of the human body. Programmed cell death mainly includes three forms: apoptosis, autophagy, and programmatic necrosis^[64]^. Since miRNAs and circRNAs can regulate cell death, the combination of drugs and miRNAs/circRNAs has been widely studied in anticancer therapy. It was reported that miR-21-5p can downregulate the expression of programmed cell death 4 (PDCD4), which leads to paclitaxel resistance^[65]^. miR-512-3p directly targets the 3’UTR of Livin, which enhances the antitumor effect of epirubicin, gemcitabine, and docetaxel^[66]^. In addition, the low expression of circ_0006528 can alleviate ADM resistance and reduce the proliferation, migration, and autoplastic apoptosis of BC cells^[67]^. Circ-ABCB10 mediated PTX resistance and apoptosis in BC cells via the let-7a-5p/DUSP7 axis^[68]^. Therefore, identifying miRNAs and circRNAs that regulate pro-apoptotic or anti-apoptotic proteins to promote apoptosis and reduce resistance to anticancer drugs in cancer cells is a prospective study.

Another mechanism of chemotherapy resistance involves adenosine triphosphate (ATP)-dependent efflux pumps that reduce intracellular drug concentrations. ATP binding cassettes (ABCs) are a class of transmembrane transporters, such as BC resistance protein (BCRP), multidrug resistance-associated protein 1 (MRP1), and P-glycoprotein (P-gp)^[69]^. There have been some studies on the regulation of various ABC transporters in acquired chemotherapy-resistant cells by miRNA and circRNA. MiR-451 can downregulate the expression of BCRP, MRP1, and P-gp, thereby reversing the resistance of BC cells to ADM^[70]^. Knockdown of circ-CHI3L1.2 downregulated the expression levels of P-GP, MRP1, and glutathione S-transferase P1 (GSTP1), and impaired cisplatin resistance^[71]^. Overexpression of P-gp reversed circRNA_103615 silencing on cisplatin resistance^[72]^. In conclusion, exploring the relationship between miRNAs/circRNAs, ABC efflux transporter proteins, and anticancer drug resistance is crucial in the search for new cancer therapeutic targets.

BC is composed of heterogeneous cell populations, which are mainly divided into two types of cell populations: BC stem cells (BCSC) and differentiated cells^[58]^. EMT is the most important pathway involved in BCSC phenotypic regulation. EMT is characterized by the loss of epithelial phenotype and the gain of mesenchymal characteristics, and it is a key factor for the development of resistance to multiple chemotherapeutic drugs. In recent years, more and more reports have shown that miRNAs and circRNAs are involved in drug resistance by regulating EMT and stem cell properties. Research found that miR-708-3p inhibits EMT and metastasis while increasing sensitivity to doxorubicin both *in vitro* and *in vivo,* by directly targeting EMT activators such as vimentin, cadherin-2 (CDH2), and zinc finger E-box binding homeobox 1 (ZEB1)^[73]^. Downregulation of miR-873 was shown to increase the mesenchymal phenotype by elevating ZEB1 expression, thereby promoting gemcitabine resistance in TNBC^[74]^. In addition, circ-PVT1 increased EMT and promoted paclitaxel resistance by inducing the expression of the regulator ZEB1 through miR-124-3p^[75]^. Therefore, EMT-inducing regulators including miRNAs and circRNAs may become useful therapeutic strategies to address drug resistance.

## MIRNAS, CIRCRNAS, AND RESPONSE TO ENDOCRINE THERAPY IN BC

Endocrine therapy is one of the commonly used treatments for BC patients and is suitable for hormone receptor-positive BC patients^[76-78]^. However, there are significant differences in the response of various patients to endocrine therapy. Recent studies have indicated that the expression of miRNA and circRNA may be closely associated with the response of BC patients to endocrine therapy. Some miRNAs and circRNAs were found to be correlated with the expression levels of endocrine receptors (ER and PR), which play a crucial role in endocrine therapy. miR-342 expression was positively correlated with ERα mRNA in tissue samples, and its introduction into ER-dependent cells enhanced BC sensitivity to tamoxifen^[79]^. circPVT1 promotes the expression of ESR1 and ERα through miR-181a-2-3p^[80]^. Antisense oligonucleotides (ASOs) targeting circPVT1 inhibited the growth of ERα-positive BC cells and tumors and sensitized tamoxifen-resistant ERα-positive BC cells to tamoxifen treatment. In addition, some miRNAs and circRNAs may also affect the sensitivity of BC cells to hormones by regulating signaling pathways or target genes related to endocrine therapy. miR-519a is a novel oncogenic miR in ER-positive BC cells that enhances cell viability, promotes cell cycle progression, and confers resistance to tamoxifen-induced apoptosis^[81]^. miR-125a-3p is downregulated in tamoxifen-resistant BC cells. It targets cell cycle-dependent kinase 3 both *in vivo* and *in vitro* and inhibits the transcriptional activity of ERα, thereby inhibiting the proliferation of ER-positive BC cells. This leads to cell cycle arrest in G1/S phase, induction of apoptosis, and inhibition of tumor growth^[82]^. CircRNA_0044556 reduces the sensitivity of TNBC cells to ADM through the miR-145/NRAS axis^[83]^. Through an in-depth investigation of the mechanisms of action of miRNA and circRNA in endocrine therapy, we hope to identify new biomarkers for predicting the response of BC patients to endocrine therapy and provide a basis for personalized treatment. These research findings may contribute to improving the treatment outcomes of BC patients, reducing unnecessary treatments, and promoting the development of personalized and precision medicine in BC therapy.

## MIRNAS, CIRCRNAS, AND RESPONSE TO TARGETED THERAPY IN BC

Targeted therapy is a cancer treatment method that targets specific genes or proteins. Trastuzumab is the first humanized mAb developed for HER2 and has achieved remarkable success in the treatment of HER2-positive BC^[84]^. However, it has been observed that some BC patients are insensitive to HER2 treatment or change from susceptible to resistant. Increased expression of miR-221 can be observed in trastuzumab-resistant cells^[85]^. Circ-BGN is overexpressed in trastuzumab-resistant BC tissues, and its downregulation reduces cell viability, especially restoring sensitivity to trastuzumab^[86]^. These findings indicate that the expression of miRNA and circRNA has an impact on the efficacy of trastuzumab. In addition, overexpression of circCDYL2 stabilized growth factor receptor-bound protein 7 (GRB7) by preventing its ubiquitination degradation, thereby making BC cells resistant to trastuzumab^[87]^. Therefore, an in-depth exploration of the mechanisms of miRNA and circRNA in the response to targeted therapies could be helpful for BC-targeted therapies.

## CONCLUSION

**Figure 4:**
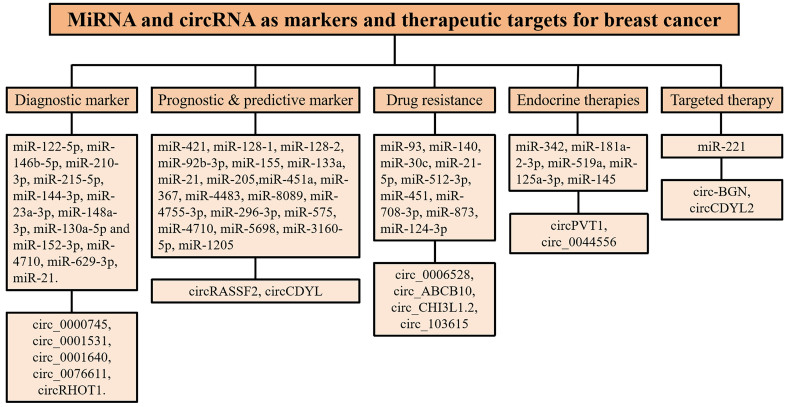
miRNA and circRNA as markers and therapeutic targets for BC. BC: Breast cancer.

BC ranks first in global incidence and mortality among female cancers. Identifying key biomarkers in the progression of BC can address the urgent need to enhance personalized treatment and improve the cure rate. MiRNA is abnormally expressed in many cancers, and can be utilized for the diagnosis and classification of BC. MiRNA data are easy to obtain, sensitive, and reliable, which can inspire research on the occurrence and progression of BC. With the advancement of miRNA chip and deep sequencing technologies, new miRNA biomarkers have been identified [Figure 4]. By interacting with miRNAs and other molecular effectors, circRNAs modulate gene expression at various levels, contributing to the intricate network of dysregulated signaling pathways in cancer. In BC, aberrant circRNA expression profiles have been implicated in tumorigenesis, progression, and metastasis [Figure 4]. Importantly, several dysregulated circRNAs show potential as diagnostic and prognostic markers, highlighting their valuable utility as clinical tools for BC management.

Despite significant progress in understanding the role of miRNA and circRNA in BC, challenges and opportunities remain. First, due to the large differences in the number of enrolled samples, current research on miRNA and circRNA as biomarkers still faces obstacles related to inconsistency and irreproducibility. Unifying the requirements for patient inclusion and the number of selected cases will be the key to solving this problem. Second, due to the limited sensitivity of current technologies and the specific objectives of researchers, discrepancies exist in studies assessing the association between miRNA and circRNA and their target genes. It is necessary to combine extensive multicenter studies with basic research to better characterize specific miRNAs and circRNAs and their related pathways. Finally, current therapeutic research on miRNA and circRNA is mainly at the preclinical stage, and miRNA and circRNA mimics or antagonists are mainly used to promote or inhibit their roles in BC. Addressing issues such as poor transfection efficiency and off-target effect is crucial.

Exploring the potential of miRNAs and circRNAs as diagnostic and prognostic biomarkers for BC represents a paradigm shift in the field of oncology, offering possibilities for more precise and personalized diagnostic and therapeutic strategies for BC. By leveraging the unique expression profiles of these non-coding RNAs, clinicians may soon be able to stratify patients according to their molecular characteristics to guide treatment decisions and improve patient outcomes. Furthermore, the development of innovative technologies for the detection and quantification of miRNAs and circRNAs holds promise for implementing non-invasive and cost-effective screening strategies, particularly in resource-limited settings. In conclusion, research on miRNAs and circRNAs may lead to significant changes in the management of BC patients and improve diagnosis and prognosis.

## DECLARATIONS

### Copyright

© The Author(s) 2024.
